# Dissemination and implementation science activities across the Clinical Translational Science Award (CTSA) Consortium: Report from a survey of CTSA leaders

**DOI:** 10.1017/cts.2019.422

**Published:** 2019-09-25

**Authors:** Rowena J. Dolor, Enola Proctor, Kathleen R. Stevens, Leslie R. Boone, Paul Meissner, Laura-Mae Baldwin

**Affiliations:** 1 Department of Medicine, Division of General Internal Medicine, Duke University, Durham, NC, USA; 2 Brown School, Washington University in St. Louis, St. Louis, MO, USA; 3 School of Nursing, University of Texas Health San Antonio, San Antonio, TX, USA; 4 Vanderbilt Institute for Clinical and Translational Research, Vanderbilt University Medical Center, Nashville, TN, USA; 5 Office of the Medical Director for Research, Montefiore Medical Center/Albert Einstein College of Medicine, The Bronx, NY, USA; 6 Department of Family Medicine and Institute of Translational Health Sciences, University of Washington, Seattle, WA, USA

**Keywords:** Dissemination, implementation, CTSA, translational science, NIH, research training, workforce development

## Abstract

**Introduction:**

Dissemination and implementation (D&I) science is not a formal element of the Clinical Translational Science Award (CTSA) Program, and D&I science activities across the CTSA Consortium are largely unknown.

**Methods:**

The CTSA Dissemination, Implementation, and Knowledge Translation Working Group surveyed CTSA leaders to explore D&I science-related activities, barriers, and needed supports, then conducted univariate and qualitative analyses of the data.

**Results:**

Out of 67 CTSA leaders, 55.2% responded. CTSAs reported *directly funding* D&I programs (54.1%), training (51.4%), and projects (59.5%). *Indirect support* (e.g., promoted by CTSA without direct funding) for D&I activities was higher – programs (70.3%), training (64.9%), and projects (54.1%). Top barriers included funding (39.4%), limited D&I science faculty (30.3%), and lack of D&I science understanding (27.3%). Respondents (63.4%) noted the importance of D&I training and recommended coordination of D&I activities across CTSAs hubs (33.3%).

**Conclusion:**

These findings should guide CTSA leadership in efforts to raise awareness and advance the role of D&I science in improving population health.

## Introduction

The Institute of Medicine has reported a delay of up to 17 years between the generation of new medical discoveries and their translation into clinical practice [1–2]. Dissemination and implementation (D&I) science is critical to speeding the translation of evidence-based discovery into practice in order to make more rapid improvements in human health.

Currently, D&I science is not a formalized element of the Clinical Translational Science Award (CTSA) Program. However, individual CTSAs have recognized the importance of D&I science resources to the clinical translational science enterprise. Of the 45 CTSA institutions that participated in the 2010 CTSA Comparative Effectiveness Research Needs and Capacity Assessment Survey asking about the extent to which their institution needed to increase capacity and activity in D&I research, 54.5% reported “to some extent” and 43.2% reported “to a large extent” (CTSA Comparative Effectiveness Research Key Function Committee, unpublished data). CTSA recognition of the importance of D&I science has been spurred further by the 2013 assessment of CTSA program accomplishments, which pointed to the need for expanded activities to engage clinical stakeholders and move research into practice [2]. Finally, the National Center for Advancing Translational Strategic Plan includes implementation research in its Translational Science Spectrum [3]. Federal agencies such as the National Institutes of Health (NIH), the Agency for Healthcare Research and Quality, and the Veterans Administration have all responded with calls for D&I research by increasing the number of funding opportunities. In addition, there are growing solicitations for D&I research from nonfederal sources, such as the Patient-Centered Outcomes Research Institute (PCORI). Although PCORI primarily funds comparative effectiveness research, they have added specific funding opportunities for D&I research for former awardees to spread their innovations beyond the original project.

Some CTSA hubs have reported their support for D&I science activities. The Washington University CTSA’s Dissemination and Implementation Research Core describes their institution’s broader infrastructure for D&I research, and CTSAs are referenced in work identifying D&I science training opportunities [4–5]. Morrato et al. conducted key informant interviews with CTSA institution representatives about their practices and opportunities for improving national comparative effectiveness research translation through D&I science via CTSA institutions and made three recommendations for bolstering D&I science in these institutions: (1) create a national clearinghouse for D&I science tools, (2) identify sources for best D&I science practices, and (3) help network CTSA institutions with existing D&I science resources [6]. To do so, identifying D&I science resources, training, and research efforts across the CTSA Consortium, as well as better understanding factors that challenge and promote their provision across the 64 CTSA programs is critical, but is largely unknown.

To fill this knowledge gap, the Dissemination, Implementation, and Knowledge Translation (DI&KT) Science Working Group within the CTSA program is identifying emerging, as well as well-developed resources, programs, training/workforce development efforts, and scientific research projects related to D&I science that are directly funded by CTSA programs. The working group is also identifying whether CTSAs are supporting other D&I science activities or collaborating with other groups conducting D&I science activities within their institutions, even if they are not directly funding these activities. Here we report on a survey of the 64 CTSA Principal Investigators (PIs) and Administrative Directors about their programs’ current D&I science activities, and their experience with providing these resources as part of their CTSA programs.

## Methods

For the purposes of this study, we defined D&I science as the scientific study of the use of strategies to adopt, integrate, and spread evidence-based health interventions into clinical and community settings in order to improve patient outcomes and benefit population health.

The DI&KT Working Group created a short seven-question survey asking the following:

- Whether the CTSA directly funds (partially or fully) (1) D&I science programs/resources, (2) D&I science trainings/workforce development, or (3) D&I scientific research projects;
- Whether the CTSA collaborates or supports (without use of CTSA funds) the same three D&I science activities listed above;
- If D&I science activities are funded, the challenges or barriers they encountered in developing and supporting D&I science activities within their CTSA;
- If no D&I science activities are funded by the CTSA, the reasons why;
- To list up to three things that would help their CTSA program in supporting researchers to include D&I science activities across all phase of research (besides funding);
- To identify three existing services/resources available to the larger CTSA Consortium that can be used more strategically to support D&I science within the overall CTSA program.
- The name and contact information of person(s) involved in D&I science who could provide detailed information about D&I activities at their institution.

Direct support was defined as CTSA-allocated funds (partial or full) for D&I science activities. Indirect support was defined as promoting and/or collaborating on D&I science activities occurring within their institution that are not funded by the CTSA award. Examples of the three types of D&I science activities included:

- *D&I science program/resource:* D&I Research Core, D&I Consultation services;
- *D&I science training/workforce development:* Training course or workshop on implementation science;
- *D&I scientific research project:* Pilot funding for D&I research project, development of methods/measures for implementation research.

Because we wanted to focus on scientific activities specific to D&I, the survey instructions clearly stated not to include the existence of a Community Engagement core as a D&I science activity, and not to list the following applied D&I activities: (1) contacting the media about important findings from a CTSA-sponsored pilot project; (2) distributing a newsletter about CTSA activities; (3) hosting a lecture series; (4) conducting a CTSA-sponsored workshop on how to publish research findings; or (5) collaborating with another CTSA hub to distribute a new tool for implementation of evidence-based practices.

The survey was reviewed and revised by the CTSA Collaboration and Engagement Domain Task Force Lead Team, then by the CTSA PI Steering Committee. After approval from the CTSA program leaders at the National Center for Advancing Translational Science, the survey was programmed into REDCap and sent to the CTSA PIs and the Administrative Directors. Two e-mail reminders were sent to the CTSA leaders, and an additional reminder was inserted in the CTSA newsletter on July 14, 2017. Survey responses were collected between June 6 and August 18, 2017 and exported into Microsoft Excel for analysis. If more than one response was received from a single institution, we counted that institution once for the denominator, but combined the write-in information collected from all of the respondents. For example, on the yes/no questions, if one respondent marked “yes” to having a resource and another person marked “no,” then we listed the response as “yes.” For the write-in questions, we counted redundant answers only once (e.g., funding challenges), but retained all unique responses. Therefore, we have complete information from all surveys collected from each institution.

We calculated rates/proportions for the yes/no questions. Two investigators (LMB, RJD) reviewed the data from the survey’s open-ended questions and coded these into common themes. One investigator did the initial coding, and the other investigator verified the coding. Discrepancies were reviewed and consensus was used to determine the final coding.

We obtained information on individual CTSA programs’ direct cost and number of years of CTSA funding from NIH RePORTer [7] and compared average direct cost and years as a CTSA between respondent and nonrespondent CTSAs. We assigned CTSA programs by state to US Census Regions using definitions published by the US Census and compared distribution of respondent and nonrespondent CTSAs by region [8].

## Results

Of the 67 CTSA leaders surveyed, 37 responded (55.2% response rate). Supplemental Table 1 compares the award size, geography, and years as a CTSA between respondent and nonrespondent CTSAs. The median direct costs of the CTSA award for fiscal year 2017 among respondent and nonrespondent CTSAs were nearly the same – $3,639,047 and $3,829,500, respectively. The greatest proportion of respondent CTSAs was in the West and Midwest Census Regions (51.3%), and in the South and Northwest for nonrespondent CTSAs (66.6%). The average length of time as a CTSA member institution was 9 years for both groups. Overall, there were no significant differences in these characteristics between the two groups.

### Support for D&I Science Activities

Table 1 summarizes the direct and indirect support of CTSAs for the three types of D&I science activities. Of the 37 respondents, 3 CTSA institutions reported no direct or indirect support for any D&I science activities. About half of responding CTSAs reported directly funding each of the three types of D&I science activities: research programs/resources (54.1%), training/workforce development (51.4%), and scientific research projects (59.5%). Indirect support for D&I activities was somewhat higher among responding CTSAs – 70.3% supported D&I science programs/resources, 64.9% training/workforce development, and 54.1% scientific research projects.

**Table 1.** Proportion of respondent Clinical Translational Science Awards (CTSA) directly and/or indirectly supporting dissemination and implementation (D&I) activities

	D&I science program/resource^a^ *N* (%)	D&I science training/workforce development *N* (%)	D&I scientific research projects^b^ *N* (%)
Direct CTSA funding (*n* = 37)	20 (54.1)	19 (51.4)	22 (59.5)
Indirect CTSA support (*n* = 37)	26 (70.3)	24 (64.9)	20 (54.1)
Either type of support (*n* = 37)	28 (75.7)	26 (70.3)	28 (75.7)

a Examples: D&I Research Core, consultation services for design of D&I research projects.

b Examples: Development of outcome measures for implementation science, pilot funding for D&I science projects.

The majority of CTSAs directly funded at least one D&I science activity (70.3%), but only 35.1% directly funded all three activities (Supplemental Table 2). The vast majority of responding CTSAs indirectly funded at least one D&I science activity (81.1%), and 43.2% indirectly supported all three activities.

### Challenges/Barriers to D&I Science Support

Open-ended questions were included to allow respondents to name three challenges or barriers to developing and supporting D&I science activities within their CTSA. Table 2 summarizes the results by theme. Funding was the most frequently cited barrier (39.4%). Funding barriers included lack of funding to protect faculty time for working on D&I science programs, limited funds for pilot studies, and fewer external grant opportunities for D&I science. Several CTSAs (30.3%) mentioned the limited number of faculty adequately trained to lead D&I science programs and training, and to mentor young investigators interested in D&I science. Some CTSAs (27.3%) commented that faculty have a lack of understanding of D&I science and the resources available, as well as noted a perception that D&I science is not a well-defined area and thus more difficult to fit into CTSA programs.

**Table 2.** Challenges/Barriers to developing and supporting dissemination and implementation (D&I) science activities reported by Clinical Translational Science Awards (CTSA)

Challenges/Barriers	CTSAs reporting *N* (%) (*n* = 33^a^)
Funding	13 (39.4)
Inadequate D&I science workforce	10 (30.3)
Lack of understanding of D&I science	9 (27.3)
Engagement and competing priorities of clinician workforce/health systems	6 (18.2)
Inexperience with D&I science Inadequate training curriculum/resources Culture does not support D&I science activities	3 (9.1)
Inadequate IT resources to support D&I science Lack of D&I science mentors Funding for D&I science separate from Community Engagement Lack of awareness of D&I science resources	2 (6.1)
Hard to find fit in current CTSA structure Clinical tools or Health Information Technology to facilitate D&I science exist, but not integrated into research Lesser priority in CTSA; not prioritized in CTSA request for application	1 (3.0)

a Four institutions did not reply to this question.

### Needed Supports for D&I Science

Respondent CTSAs were asked to name three things (not including funding) that would help them develop and support D&I science activities within their CTSA. Table 3 summarizes the results by theme. A majority of respondents (63.4%) noted the importance of D&I science training activities, especially in D&I science methods and best practices, as well as in how D&I science can contribute to research across the translational spectrum. Growing the D&I science workforce (30.3%), in particular mentors, was another important strategy for helping CTSAs support researchers to include D&I science activities within all phases of translational research. National coordination across CTSA D&I science programs (24.2%), tools and resources to support the use of best practice D&I science methods (21.2%), and consultation services (12.1%) were related to strategies for supporting researchers to include D&I science in their research.

**Table 3.** How to help Clinical Translational Science Awards (CTSA) support researchers to include dissemination and implementation (D&I) science activities across all phases of research (excluding funding)

How to help CTSAs support researchers to include D&I science activities	CTSAs reporting *N* (%) (*n* = 33^a^)
Training	21 (63.6)
More D&I science workforce	10 (30.3)
National coordination	8 (24.2)
Tools and resources	7 (21.2)
Greater visibility/awareness of D&I science Methods	5 (15.2)
Consultation service Health system engagement	4 (12.1)
Allocation of CTSA resources Better dissemination platforms Clear National Center for Advancing Translational Science (NCATS)/ National Institutes of Health (NIH) mandates	2 (6.1)
Informatics support Training peer reviewers D&I science core in the CTSA Leverage parent institution’s resources	1 (3.0)

a 4 institutions did not reply to this question.

### CTSA Consortium Resources for D&I Science

Based on the existing services and/or resources available to the larger CTSA Consortium, respondents were asked to identify three services and/or resources that can be used more strategically to support D&I science. One-third of respondents expressed that the CTSA program could more strategically coordinate D&I science activities across CTSAs hubs and support collaboration among hubs (Table 4). Several cited the need to create a compendium of D&I science educational materials and to provide trainings (27.3%) as well as to have D&I science resources and tools (24.3%) for CTSAs to share widely at their institutions.

**Table 4.** Clinical Translational Science Award (CTSA) Consortium services and resources that can be used strategically to support dissemination and implementation (D&I) science

Supportive CTSA Consortium services and resources	CTSAs reporting *N* (%) (*n* = 33^a^)
Collaboration across CTSAs	11 (33.3)
Educational materials/training	9 (27.3)
Tools/resources	8 (24.3)
CTSA training programs (e.g., KL2, TWD) Lack of awareness of CTSA Consortium resources	6 (18.2)
CTSA Community Engagement core	4 (12.1)
Pilots Other/Collaborations outside CTSA	3 (9.1)
Cross CTSA mentorship Evaluation Informatics, especially related to health care systems Trial Innovation Network Funding	2 (6.1)
Biostatistics, epidemiology and research design D&I science theme groups Common metrics Multi-hub studies	1 (3.0)

KL2, a type of mentored career development award; TWD, Training and Workforce Development.

a 4 institutions did not reply to this question.

## Discussion

The current survey shows that, 7 years after the 2010 survey of CTSA PI’s who endorsed the need to increase their capacity and activity in D&I research [6], just over half of responding CTSAs directly funded at least one D&I science resource or consultation program, training or workforce development effort, or scientific research project. Roughly 70% indirectly supported or promoted at least one D&I science activity at or affiliated with their CTSA’s institution.

At the time of this survey, only about a third of CTSAs (35.1%) had a complete package of directly funded D&I science activities, including consultation/resources, training/workforce development, and funding opportunities. Almost a third (29.7%) of all respondent CTSA programs did not directly support any D&I science activities, representing a significant gap in support for translational science resources. It is likely that nonrespondent CTSAs would have an even lower rate of directly funding these D&I science activities.

These CTSAs reported that a robust D&I science-experienced workforce, D&I training, and D&I tools were the top resources needed to overcome challenges and barriers to building capacity for D&I science at their institutions. Responding CTSA leaders felt that the CTSA Consortium could help fill this gap by offering training, especially in D&I science methods and best practices for new investigators. This is critical because the current demand for D&I science training outstrips the available programs [5]. One recommendation was to develop a training exchange between a CTSA with content expertise and training opportunities and a CTSA without this expertise. Another strategy that the CTSA program could use to increase D&I science training and workforce development is to include D&I science competencies within the core competencies for clinical translational science [9]. A number of training opportunities in D&I science have recently emerged from immersive training institutes to webinar series to career development awards, many in institutions with CTSA hubs [10]. CTSA hubs can ensure that knowledge of these opportunities is disseminated widely among their stakeholders and that appropriate opportunities are targeted to investigators at different career stages. Additionally, CTSA hubs can benefit from the collaborative efforts of experts from several of these training programs to identify how to best meet the needs of diverse trainees [5, 10]. All of these opportunities are important to growing the D&I science workforce and creating more mentors who can support D&I science learners.

Engagement and competing priorities of the clinician workforce and health system leadership were noted by respondents as a barrier to implementation science. Existing clinical initiatives (e.g., quality improvement projects) within health systems may serve as barriers to clinician and practice participation in research. Concerns about reducing clinical revenue as a result of research implementation may decrease practice participation. Designing D&I science projects that complement health system priorities, are pragmatic for busy clinical environments, and offer fair compensation to practices for the time spent on projects are all attributes that can challenge D&I researchers. At the same time, designing D&I research that fits with health system priorities is both a necessity and opportunity for the field of D&I science and its collaborating health care institutions. As one example, partnership between D&I researchers and health systems has potential to facilitate health system transformation into learning health systems.

Perhaps the most significant finding is that there is a reported lack of understanding of D&I science across the CTSA Consortium. This is the first time this has been explicitly identified and suggests that D&I scientists have work to do in communicating and “disseminating” the value of D&I science to the translational science community. The DI&KT Working Group sponsored a well-attended national webinar [11] entitled “Dissemination and Implementation Science: What is it and Why is it critical to Translational Science?” which addressed the importance of D&I science to translational research. The webinar offered an example of a project that progressed from proving intervention efficacy to evaluating its effectiveness in another setting and finally to its D&I in real-world settings for sustainability [12–15]. This model could be used to create a D&I science series with presentations by experts from across the CTSA Consortium.

Many PIs reported that the CTSA Consortium could play a critical role in supporting D&I science efforts in training and workforce development, provision of D&I science resources and tools, and mentorship. Ideas generated by the CTSA PIs for collaborative CTSA Consortium activities included a common portal for D&I science information and knowledge, as well as coordination of D&I science activities across the Consortium. There are CTSAs that have made substantial investments in their D&I science programs and could lead the Consortium in these collaborative efforts. Given that discoveries are unable to impact the health of the population without successful implementation and broad dissemination, establishing a model of D&I science hubs that could offer expertise to other CTSA programs may be a strategy for multiplying the effect of a currently limited D&I science workforce.

The CTSA program has taken a first step toward Consortium-wide D&I science activities by supporting the creation of the DI&KT Working Group within the Collaboration Engagement Domain Task Force in September 2016. The working group currently has 56 members from 29 CTSA institutions working to: (1) increase awareness of the critical importance of D&I science to the translation science process, (2) promote the coordination of D&I science efforts across CTSA hubs, and (3) enhance availability of D&I science methodology across CTSA settings. Members are self-identified, there is no limit to the number of members from each institution, and non-CTSA affiliated members can join. The working group has met monthly via web-enabled conference call from September 2016 until December 2019 with support from the CTSA Coordinating Centers at Vanderbilt University (September 2016–October 2017) and University of Rochester (November 2017–December 2019). To increase engagement of CTSAs in D&I science and practice, the working group has conducted the following activities, addressing some of the needs and barriers reported in the survey: (1) hosted a national webinar to increase awareness of D&I science; (2) pilot-tested metrics for D&I science activities across several CTSA institutions; and (3) developed and pilot tested a tool that CTSAs could use to document and track their D&I science consultations.

Another recommendation for supporting D&I science was for the CTSA program to provide a clear expectation to the CTSA hubs that D&I science is a critical component of translational science. This could be accomplished by establishing a mandated D&I science “core” with its own funding in the CTSA request for applications. The D&I science core could offer consultative services, training opportunities, mentorship, methodological research, and support for D&I science projects.

This survey of CTSA leaders has several limitations. First, it had a response rate of only about 58%. If we assume that many CTSAs may not have responded because they do not have active D&I science programs or activities, the gaps that we identified in D&I science training, resources, and research support could become even wider. Second, because several respondents shared that there is a lack of understanding of D&I science, it is possible that D&I science activities, especially those not directly supported by CTSA programs, may have been underreported. Third, we intentionally excluded traditional D&I activities, such as newsletters, lecture series, press releases, or collaborations among CTSA hubs to spread evidence-based practices in order to focus solely on activities related to D&I science. We assumed that all the CTSAs are active in these traditional dissemination activities. The goal of the survey was to identify gaps in scientific D&I activity, such as consultation, training, mentorship, and grant opportunities. Finally, this short survey provides only a simple snapshot of the support that CTSA hubs provide for D&I science activities. In a second phase of the DI&KT Working Group’s environmental scan, we surveyed contacts that the PIs referred to as local D&I science experts to obtain more detailed information about the D&I science resources, training, and projects at their institutions (results under analysis). This more detailed survey will allow us to understand the depth with which the CTSA program is currently supporting D&I science activities.

The CTSA Consortium is designed “to improve the translational research process to get more treatments to more patients more quickly” [16]. This survey has demonstrated that the CTSA Consortium has the opportunity to take a leadership role in developing and supporting the D&I science that is critical throughout the translational spectrum to ensure that treatments reach patients most effectively and efficiently. Currently, CTSA leaders report that CTSA Consortium contributions to D&I science have been limited by a dearth of D&I science-trained workforce members, low funding levels for D&I science, and a lack of understanding of D&I science and its role in furthering CTSA objectives. The survey findings that the DI&KT Working Group presents here can be used to support and guide the CTSA program and its Consortium to recognize the CTSA program’s critical role in leading the advancement of the science of D&I to improve population health nationally.

## Supplementary material

For supplementary material accompanying this paper visit https://doi.org/10.1017/cts.2019.422. click here to view supplementary material

## Appendix: DI&KT Working Group members

**Table:**

Last name	First name	University name
Auyoung	Mona	Scripps Research Institute
Baldwin	Laura-Mae	University of Washington
Baumann	Ana	Washington University in St. Louis
Bender	Miriam	University of California, Irvine
Bennett	Nancy	University of Rochester
Chambers	David	National Institutes of Health
Clark	Robert	University of Texas Health Science Center at San Antonio
Cole	Allison	University of Washington
Connors	Katherine	Stanford University
Cooper	Dan	University of Chicago at Illinois
Corbin	Keesha	Medical University of South Carolina
Daudelin	Denise	Tufts University
Dickinson	Miriam	University of Colorado Denver
Dolor	Rowena	Duke University
Eliacin	Johanne	Indiana University
Fernandez	Maria	University of Texas Health Science Center at Houston
Foley	Kristie	Wake Forest University
Handley	Margaret	University of California, San Francisco
Holtrop	Jodi	University of Colorado Denver
Jones	Patricia	National Center for Advancing Translational Sciences
Kasper	Amanda	Children’s Research Institute
Kripalani	Sunil	Vanderbilt University Medical Center
Kwan	Bethany	University of Colorado Denver
Kwon	Simona	New York University School of Medicine
Leppin	Aaron	Mayo Clinic
Mahoney	Jane	University of Wisconsin-Madison
Malcolm	Elizabeth	Duke University
McGovern	Mark	Stanford University
McGuire	Alan	Indiana University
Mehta	Tara	University of Illinois at Chicago
Meissner	Paul	Albert Einstein College of Medicine
Melvin	Cathy	Medical University of South Carolina
Meyer	Jessica	Stanford University
Miller	David	Wake Forest University
Mittman	Brian	University of California, Los Angeles
Montori	Victor	Mayo Clinic
Moore	Justin	Wake Forest University
Morrato	Elaine	University of Colorado Denver
Nease	Donald	University of Colorado Denver
Parchman	Michael	MacColl Center for Health Care Innovation
Patel	Sapana	Columbia University
Patino-Sutton	Cecilia	University of Southern California
Pincus	Harold	Columbia University
Proctor	Enola	Washington University in St. Louis
Purcell	Kathy	University of Wisconsin-Madison
Quanbeck	Andrew	University of Wisconsin-Madison
Reistetter	Tim	University of Texas Medical Branch Galveston
Robinson	Christina	Children’s Research Institute
Rohweder	Catherine	University of North Carolina
Scholl	Linda	University of Wisconsin-Madison
Shah	Nilay	Mayo Clinic
Shea	Christopher	University of North Carolina
Shelton	Rachel	Columbia University
Shenkman	Elizabeth	University of Florida
Singh	Indrani	Center for Leading Innovation & Collaboration
Smith	J.D.	Northwestern University
Smith	Maureen	University of Wisconsin-Madison
Stadnick	Nicole	University of California, San Diego
Stevens	Kathleen	University of Texas Health Science Center at San Antonio
Taras	Howard	University of California, San Diego
Tobin	Jonathan	Clinical Directors Network
Turner	Barbara	University of Southern California (current); University of Texas Health Science Center at San Antonio (former)
Walkey	Allan	Boston University
Weiner	Bryan	University of Washington
Weis	Jennifer	Mayo Clinic
Whitcomb	Pam	Center for Leading Innovation & Collaboration
Zender	Robynn	University of California, Irvine
