# Supplementary material for: Dissemination and implementation science activities across the Clinical Translational Science Award (CTSA) Consortium: Report from a survey of CTSA leaders
Source: J Clin Transl Sci. 2019 Sep 25;4(3):188–94. doi: 10.1017/cts.2019.422 (PMC7348014; doi:10.1017/cts.2019.422)
Supplement: Supplementary file 1 [file S2059866119004229sup001.docx]

**Supplemental Table 1: Characteristics of Respondent and Nonrespondent CTSA Programs**

| **Characteristic** | **Respondents**  **N=37** | **Non-respondents**  **N=27** |
| --- | --- | --- |
| **Average direct costs, FY 2017**  **(Standard deviation)**  **Median direct costs, FY 2017** | $5,046,681  ($4,204,250)  $3,639,047 | $4,554,762  ($2,104,093)  $3,829,500 |
| **Census Regions** |  |  |
| **West** | 21.6% | 18.5% |
| **Midwest** | 29.7% | 14.8% |
| **South** | 24.3% | 37.0% |
| **Northeast** | 24.3% | 29.6% |
| **Average number of years as a CTSA (Standard deviation)** | 9 (2) | 9 (3) |

**Supplemental Table 2: Proportion of Respondent CTSAs Directly and/or Indirectly Supporting Different Quantities of D&I Activities**

|  | **Supporting 3 D&I science activities**  **N (%)** | **Supporting 2 D&I science activities**  **N (%)** | **Supporting 1 D&I science activities**  **N (%)** | **Supporting 0 D&I science activities**  **N (%)** |
| --- | --- | --- | --- | --- |
| **Direct CTSA funding (n=37)** | 13 (35.1%) | 9 (24.3%) | 4 (10.8%) | 11 (29.7%) |
| **Indirect CTSA support (n=37)** | 16 (43.2%) | 8 (21.6%) | 6 (16.2%) | 7 (18.9%) |
| **Either type of support (n=37)** | 23 (62.2%) | 6 (16.2%) | 3 (8.1%) | 5 (13.5%) |

One point for each activity listed: 1) D&I program/resource; 2) D&I training/workforce development, 3) D&I research projects.
